# Compost and humic acid amendments are a practicable solution to rehabilitate weak arid soil for higher winter field pea production

**DOI:** 10.1038/s41598-023-44921-x

**Published:** 2023-10-16

**Authors:** Sher Muhammad, Muhammad Shaukat, Muhammad Yasin, Athar Mahmood, Muhammad Mansoor Javaid, Mohammad Khalid Al-Sadoon, Aleksandra Głowacka, Mohamed A. A. Ahmed

**Affiliations:** 1 Department of Agricultural Sciences, Allama Iqbal Open University, Islamabad, 44000 Pakistan; 2 Department of Agronomy, College of Agriculture, University of Sargodha, Sargodha, 40100 Pakistan; 3 Department of Agronomy, University of Agriculture, Faisalabad, 38040 Pakistan; 4 Department of Zoology, College of Science, King Saud University, PO Box 2455, Riyadh, 11451 Kingdom of Saudi Arabia; 5 Department of Plant Cultivation Technology and Commodity Sciences, University of Life Sciences in Lublin, 13 Akademicka Street, 20-950 Lublin, Poland; 6 Plant Production Department (Horticulture - Medicinal and Aromatic Plants), Faculty of Agriculture (Saba Basha), Alexandria University, Alexandria, 21531 Egypt; 7 School of Agriculture, Yunnan University, Chenggong District, Kunming, 650091 Yunnan China

**Keywords:** Plant sciences, Plant stress responses

## Abstract

Arid soils are often weak, low in fertility, and lack essential plant nutrients. Organic amendments might be a feasible solution to counter the detrimental impact and rehabilitate weak arid soil for the growth of legumes. The study aimed to investigate how organic amendments of compost and humic acid may affect winter field pea productivity in arid soil. Over 2 years of field experiments, a range of treatments were applied, including different amounts of compost and humic acid. The results showed higher microbial carbon (C), and nitrogen (N) biomass, root length, shoot length, grains pod^−1^, and grain yield of pea, gained from the collective application of 8 Mg ha^−1^ compost and 15 kg ha^−1^ humic acid compared to all other treatments. Organic amendments increased soil microbial C density by 67.0 to 83.0% and N biomass by 46.0 to 88.0% compared with the control. The combined application of compost and humic acid increased soil microbial N biomass by 57.0 to 60.0% compared to the sole applications of compost-only and humic acid-only. It was concluded that organic amendments of 8 Mg ha^−1^ compost and 15 kg ha^−1^ humic acid in arid soil modulated microbial density, resulting in improved winter field pea productivity. This study suggests organic amendments of compost and humic acid might be a practicable solution to rehabilitate weak arid soil to grow legumes.

## Introduction

Arid and marginal soils are often plagued by nutritional deficiencies, salinity, alkalinity, and they occasionally have low levels of organic matter. A soil examination of the marginal soil revealed that phosphorus and other plant nutrients, were deficient and not readily available^1^. One highly desired characteristic of arid soils is their biological improvement. Incorporating organic matter may be a suitable solution to improve microbial dynamics under harsh, arid climatic conditions to reclaim these soils. Suitable organic amendments in the substrate mixture improved the dynamics of plant nutrition^2^.

Organic fertilizers, such as farmyard manure, compost, and humic acid, are less hazardous for overfertilization. As soil microbes convert organic matter into inorganic mineral formations, they gradually release nutrient-rich substances^3^. Organic food supplements the food shortage. It also adds vital nutrients for plant development. Compost has a substantial impact on the physical, biological, and chemical properties of soil and has a high content of nutrients that are readily accessible to plants, which are vital for plant growth^4^. By using fewer chemical fertilizers, organic sources of nutrients not only improve crop yield and food quality but also protect our ecosystem. Bio-compost is a major constituent of waste management and acts as a significant source of macro- and micronutrients. However, methodological issues make it difficult to fully understand how microbial breakdown of compost and plant development interact: (1) the assessment of microbial biomass and activity while there are live roots and developing plants; and (2) distinguishing between a plant's growth and decaying compost or leftovers^1^. Compost activity is increased by increasing microbial biomass, total organic carbon (C), concentrations of P, and accessible nitrogen (N). The effects of compost application are usually enhanced in the nearby 10 mm soil area^5^.

Compost, derived from organic wastes, can provide a sustainable source of humic acid that can be used as a plant root stimulant. It has been proposed that the refinements of humic hydrophobic interactions within the rhizosphere of maize plants could potentially unleash substances that function as auxin-like plant growth regulators and boost plant biological activities^6^. The introduction of humic acid, in the range of 250 to 1000 mg kg^−1^, to the MM360 growth medium significantly accelerated the root development of pepper and marigold, as well as the growth of strawberry roots and fruit yield. In a separate experiment, humic acids produced from vermicomposts of food waste were combined with indol acetic acid at a rate of 10–5 μM and mixed exclusively into the MM360 growth medium (at a rate of 500 mg kg^−1^). Peppers grown in humic acids derived from vermicomposts produced from food waste of food have considerably more blooming fruits than peppers grown in commercially obtained humic acids^7^. Similarly, the growth characteristics, fruiting, nutrient profiling, and rhizosphere stochiometry of guava (*Psidium guajava* L.) in grassland wet season plant-soil interaction are all impacted by the synergistic action of humic acid substances^8^. Likewise, in a field trial, the application of humic acid increased maize yield by 44.20% in Cadmium-contaminated alkaline cropland^9^.

Leguminous crops are effective in restoring soil biological, chemical, and physical characteristics through N fixation. This ultimately enhances soil quality and nutrient availability. In arid soils, legumes have been found to exhibit a dominant microbial community structure. Furthermore, incorporating organic amendments while continuously cultivating leguminous crops can increase the organic matter content of the soil. Studies have shown that, the continuous amendment of organic fertilizers and compost contributes to enhancing organic matter in the soil^10, 11^. Pea (*Pissum sativum* L.) is a popular and important leguminous vegetable worldwide and is a particularly important multipurpose legume crop grown during the winter in arid regions worldwide. The green pods of peas are rich in protein and carbohydrates, making them an important source of nutrition for both humans and animals^12, 13^.

Compost and humic acid are organic substances that are known to improve soil health and can potentially counteract negative impacts like low fertility and the weak nutritional status of arid soils. Legumes, such as winter field peas, are important crops for sustainable agriculture due to their ability to fix N and enhance soil fertility. The research objectives were to promote sustainable agricultural practices: (i) by investigating the effects of the organic amendments of compost and humic acid on the microbial density of arid soil; (ii) to determine whether organic amendments can improve winter field pea productivity in weak arid soils.

## Materials and methods

### Experiment location

Two separate field trials were performed at the National Agricultural Research Center, Islamabad (33.6701° N, 73.1261° E) on arid soil. The first experiment was executed in 2017, followed by a repeat trial in 2018. The top 15 cm layer of soil was analyzed, and it comprised 20.25, 55.65, and 24.10% of clay, silt, and sand, respectively. The soil was fairly drained, with soil pH of 7.6; bulk density of 1.23 g cm^−3^; available P of 1.5 mg kg^−1^; total potash of 140 mg kg^−1^; nitrate–N of 3.4 mg kg^−1^, and (EC) of 1.6 dS m^−1^. The microbial biomasses present in the soil were evaluated before the experiment and contained a C microbial biomass 60 µg g^−1^; a N microbial biomass 6.0 µg g^−1^ and a P microbial biomass of 7.20 µg g^−1^.

### Experimental design and treatments

Ten different treatments were employed to explore the influence of organic additions of compost and humic acid on the productivity of the winter field pea (*Pisum sativum* L.) cultivar Climax through an increase in soil microbial density. These experiments were executed in a randomized complete block design, and treatments were replicated three times. The plot size was 15.0 m^2^ (1.5 m × 10.0 m). The treatments included; CK (control; no amendments), 4CP (4 Mg ha^−1^ compost), 6CP (6 Mg ha^−1^ compost), 8CP (8 Mg ha^−1^ compost), 10HA (10 kg ha^−1^ humic acid), 12HA (12 kg ha^−1^ humic acid), 15HA (15 kg ha^−1^ humic acid), 4CP + 10HA (4 Mg ha^−1^ compost + 10 kg ha^−1^ humic acid), 6CP + 12HA (6 Mg ha^−1^ compost + 12 kg ha^−1^ humic acid), and 8CP + 15HA (8 Mg ha^−1^ compost + 15 kg ha^−1^ humic acid). The nutrient composition of the organic amendments used in experiments is given in Table 1. The compost and humic acid were well-prepared and incorporated into the soils manually at the time of seedbed preparation. Sowing in both years was done in the month of October. Whereas, all supplementary crop husbandry practices were maintained regularly across all the treatments.

**Table 1:** Nutrient composition of compost and humic acid used in the experiments.

Organic amendments	Nitrogen (mg kg^−1^)	Phosphorus (mg kg^−1^)	Potassium (mg kg^−1^)	Organic matter (%)	pH
Humic acid	1.23	11.38	0.04	–	–
Compost	1.29	0.971	186	45	5.8

### Soil microbial biomass examination

To quantify the biomass of microorganisms that produce C and N, the fumigation-extraction approach was performed^14, 15^. A 100 g sample of soil was separated into two equal portions, each weighing 25 g of oven-dried soil. One part was exposed to ethanol-free CHCl_3_ fumigation for 24 h at 25 °C. After removing the fumigant, the soil was extracted with 100 ml of 0.5 M K_2_SO_4_ for 30 min, while shaking horizontally at 200 rpm. The solution was then sieved with folded filter paper. Meanwhile, when fumigation started, an equal amount of material was taken from the non-fumigated part. Using a Dimatoc 100 automated analyzer, organic C in the solutions was quantified as CO_2_ by infrared absorption following burning at 850 °C (Dimatec, Essen, Germany). The microbiological C biomass was estimated as follows:

$$Microbial \, C \, Biomass= \frac{(EC)}{kEC}$$

where EC is the difference between organic C extracted from fumigated soil and organic C retrieved from non-fumigated soil. Whereas, kEC is a constant that has a value of 0.45^16, 17^.

By using chemo-luminescence detection (Dima-N, Dimatec) and subsequently ignition at 850 °C, the total N in the extracts was measured as activated NO_2_. The N biomass by microbes was determined by formula;

$$Microbial \, N \, Biomass= \frac{(EN)}{kEN}$$

where EN = from fumigated soil. Whereas, kEN as a constant has a value of 0.54^17, 18^.

By using the fumigation-extraction method, the microbial P biomass was assessed^18^. After the experiment each year, a 100 g soil sample was divided into three pieces, each weighing 2.5 g of oven-dried soil. The initial 2.5 g piece was fumigated using the aforementioned method, and the remainder was extracted using 50 ml of 0.5 M NaHCO_3_ (pH 8.5) with 30 min of flat shaking at 200 rpm and 15 min of centrifuging at (2000 g) and filtering (filter paper: Schleicher & Schuell 595 ½). Once fumigation started, one section that wasn't fumigated was removed. Following the addition of 25 μg P g^−1^ (0.5 ml KH_2_PO_4_), the remainder was extracted using the same procedure as samples that had not been fumigated. Photo-spectrometry at 882 nm was used to quantify phosphate, as stated by^19^.

### Plant traits measurement

Four plants from each treatment were harvested by cutting them 3 cm above the ground at the end of the experiments in both 2017 and 2018. The length of the shoots of these four plants was measured using a scale at the time of harvest. The plant's roots were extracted and thoroughly washed. The root length of plants was measured using a root length scanner. Grains per pod were collected and counted for each pod from harvested plants. Furthermore, half of the plot was harvested, and green pods were separated from the plant to determine the green pod yield.

### Soil analysis

The samples of soil were obtained to determine physiochemical properties. To determine the soil texture, a sample of 30 g soil was first treated with 7% H_2_O_2_ for two weeks. The suspension was made with NaPO_4_, which further was processed for the removal of carbonate with 10% HCl. Thereafter, the suspension was sieved by a sieve of 63 μm diameter, and the sand fraction was weighed. A pipette technique was used to determine the amount of clay and silt in the remaining sample (< 63 μm)^20^. Electrical conductivity (EC) (1:5 v/v) by EC meter and soil pH (1:2.5 v/v) by pH meter were also measured from soil–water suspension.

Using a Vario Max CN analyzer (Elementar, Hanau, Germany), the total amounts of organic C and N were calculated. By employing HNO_3_ pressure digestion and measured by ICP-AES (Spectro Analytical Instruments/Kleve) total P was measured according to^21^.

### Statistic

Separate analyses were executed on two independent field experiments for the winter field pea crops. The results are provided as the mean of three replicates and the standard deviation after being evaluated using a one-way analysis of variance. To check for significance between treatments, *p* ≤ 0.05 was used. A post hoc test was utilized to conduct all pairwise comparisons, and Tukey's HSD test was employed to evaluate significant levels with a probability threshold of 5%.

### Statement

It is confirmed that plant material used in experiments follows the guidelines and regulations as described by IUCN Policy Statement on Research Involving Species at Risk of Extinction and the Convention on the Trade in Endangered Species of Wild Fauna and Flora.

## Results

### The density of soil microbial biomass

**Figure 1:**
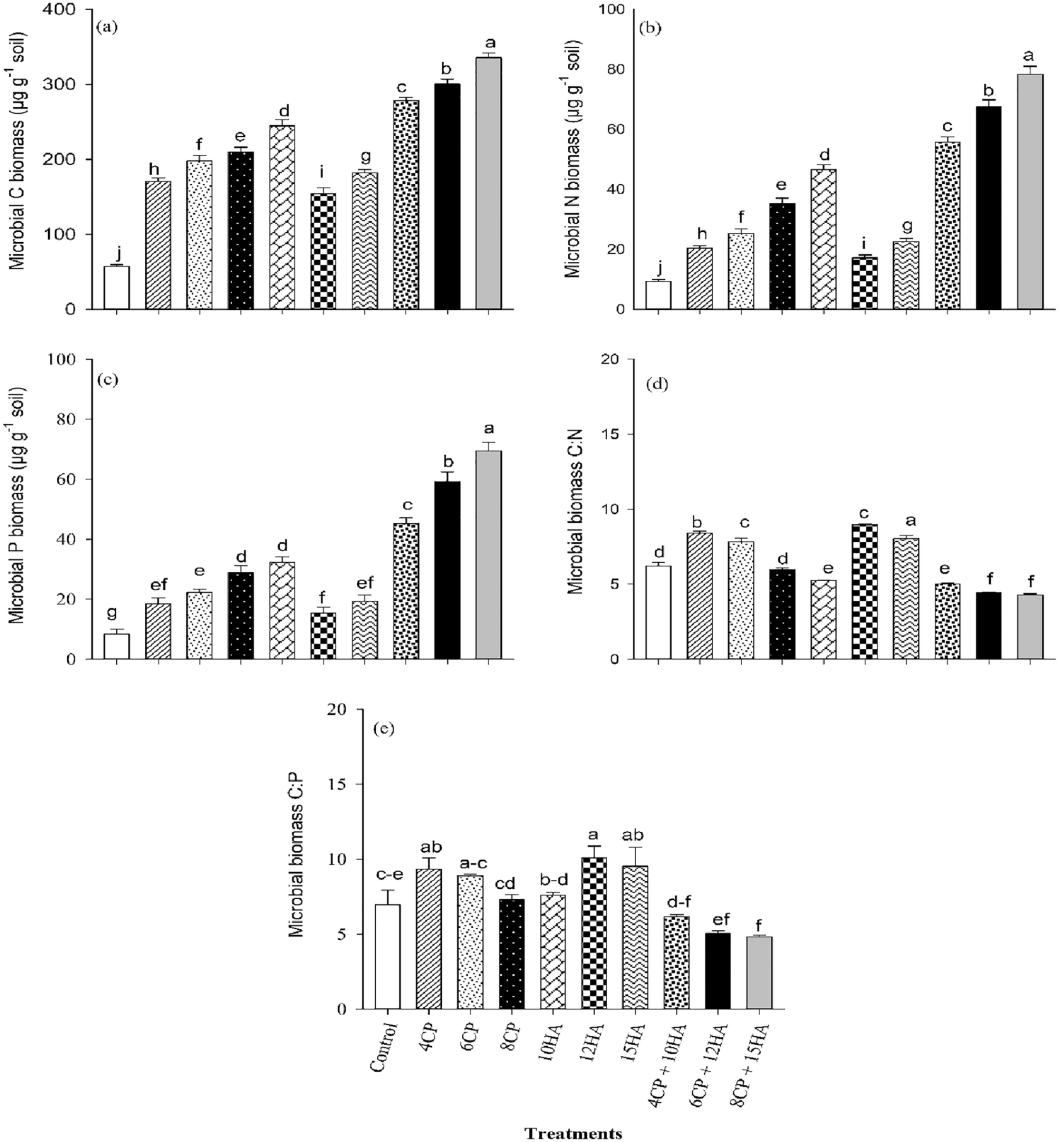
Compost and humic acid incorporation in arid soil improved the productivity of field peas by modulating soil microbial densities and biomasses. (**a**) Microbial C biomass (µg g^−1^ soil), (**b**) Microbial N biomass (µg g^−1^ soil), (**c**) Microbial P biomass (µg g^−1^ soil), (**d**) Microbial biomass C:N, (**e**) Microbial biomass C:P. The Tukey's HSD test results at *p* < 0.05. At the top of the vertical bars, distinct letters revealed apparent variances in treatment mean values. The standard deviation is displayed in the error bars (*n* = 3).

In general, the results indicated that all organic amendments had a substantial effect on arid soil microbial biomasses, including P, C, and N biomasses along with microbial C:N and C:P ratios (Fig. 1). The highest microbial C biomass was observed by incorporating 8CP + 15HA, in arid soil followed by 6CP + 12HA (Fig. 1a). Organic amendments increased microbial C density by 67.0 to 83.0% compared to CK (Fig. 1a). The amendment of compost-only and humic acid-only caused in a reduction in soil microbial C biomass compared to the combined use of compost and humic acid (Fig. 1a). Similarly, the highest soil microbial N biomass was detected in soil with 8CP + 15HA, followed by 6CP + 12HA (Fig. 1b). The organic amendments increased soil microbial N biomass by 46.0 to 88.0% relative to CK (Fig. 1b). Similarly, the combined use of compost and humic acid treatments also increased soil microbial N biomass by 57.0 to 60.0% relative to compost-only and humic acid-only (Fig. 1b). The maximum microbial P biomass was recorded from 8CP + 15HA followed by 6CP + 12HA (Fig. 1c). Organic amendments increased microbial P biomass by 55.0 to 89.0% compared to CK (Fig. 1c). Additionally, the use of compost-only and humic acid-only reduced the soil microbial P biomass by 60.0% compared with the combined use of compost and humic acid (Fig. 1c). Higher microbial biomass C:N was noticed from 15HA followed by 4CP (Fig. 1d). The combined use of compost and humic acid significantly reduced the CN ratio of microbial biomass (Fig. 1d). Subsequently, combined used of compost and humic acid also reduced the C:P of soil microbial biomass. A higher CP ratio in microbial biomass was reported from the applications of sole compost-only and sole humic acid-only (Fig. 1e).

### Field pea’s nutrients uptake and assimilation

**Figure 2:**
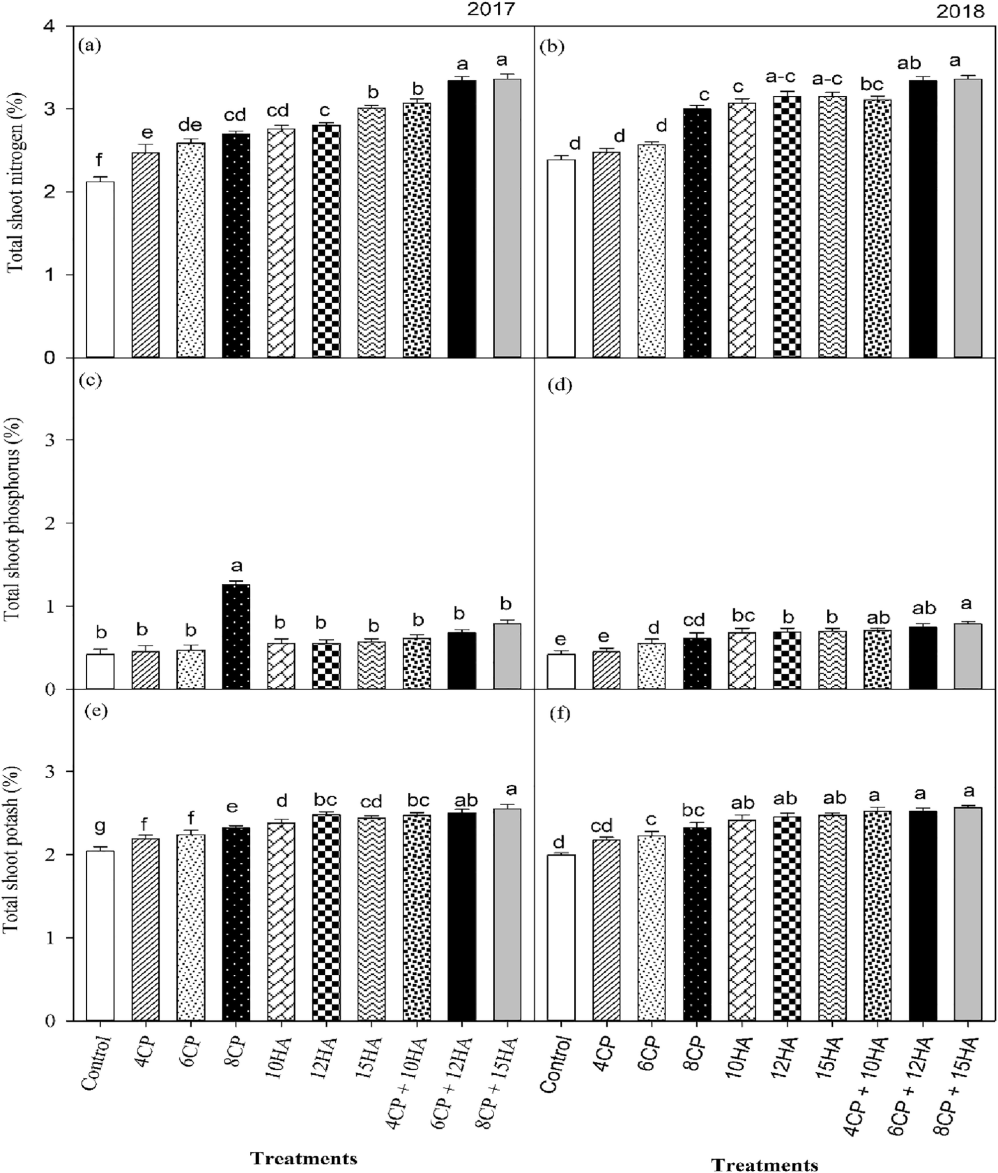
Response of compost and humic acid incorporation on the absorption and assimilation of nutrients by the field pea for the year 2017 and in repeat experiment 2018. Total shoot nitrogen % (**a**, **b**), total shoot phosphorous % (**c**, **d**), total shoot potash % (**e**, **f**). Tukey's HSD test results at *p* < 0.05. At the top of the vertical bars, distinct letters revealed apparent variances in treatment mean values. The standard deviation is displayed in the error bars (*n* = 3).

The amendments of compost and humic acid organics, had a significant influence on the nutrient composition of field peas in the 2017 and 2018 growing years (Fig. 2). In particular, the application of compost and humic acid organics enhanced shoot N contents by 11.0 to 35.0% relative to the CK, whereas the combined application of compost and humic acid enhanced shoot N content by 8.0 to 19.0% compared to the sole application of compost-only and humic acid-only (Fig. 2a,b). Furthermore, the organic amendments resulted in enhanced shoot P contents by 22.0–44.0% compared to CK (Fig. 2c,d). The combined use of compost and humic acid also enhanced shoot P contents by 12.0 to 14.0% compared to the sole application of compost-only and humic acid-only. Organic amendments significantly enhanced shoot K contents by 9.4 to 21.4% compared to CK. The combined applications of compost and humic acid increased shoot K contents by 3.2 to 11.0% as compared to the compost-alone and humic acid-alone application (Fig. 2e,f).

### Pea biomass accumulation

Organic amendments of compost and humic acid significantly improved the green pod yield and related attributes of field peas in both years (Table 2). The maximum shoot length of the pea was observed from 8CP + 15HA followed by 10HA during both growing seasons. While no significant difference in shoot length was noted among compost treatments (Table 2). In the case of root length, organic amendments produced plants with similar root lengths.

**Table 2:** The effect of organic amendments of compost and humic acid on shoot length, root length, grains pod^−1^, and green pod yield of field pea.

Treatments	Shoot length (cm)	Root length (cm)	Grains pod^−1^	Pod yield (kg ha^−1^)
Winter field pea traits (the year 2017)				
CK	78.5 ± 4.5e	15.3 ± 2.7b	4.8 ± 0.9a	612 ± 90g
4CP	94.3 ± 8.0d	21.5 ± 2.1a	5.5 ± 0.8a	930 ± 115f
6CP	95.4 ± 7.9d	20.5 ± 1.8a	7.3 ± 1.6a	1065 ± 102ef
8CP	95.7 ± 6.9d	21.3 ± 2.8a	8.3 ± 1.6a	1457 ± 135b–d
10HA	110.5 ± 5.3ab	21.6 ± 2.8a	7.7 ± 1.7a	1287 ± 87de
12HA	104.4 ± 4.4bc	20.7 ± 1.9a	8.4 ± 2.2a	1345 ± 105cd
15HA	96.7 ± 3.7d	19.6 ± 1.8ab	7.8 ± 1.8a	1330 ± 109cd
4CP + 10HA	98.4 ± 2.6cd	20.9 ± 1.8a	8.3 ± 2.2a	1540 ± 97bc
6CP + 12HA	98.3 ± 2.9cd	20.6 ± 1.9a	8.5 ± 2.2a	1680 ± 112b
8CP + 15HA	113.6 ± 4.3a	21.7 ± 1.8a	9.3 ± 2.3a	1995 ± 125a
Winter field pea traits (the year 2018)				
CK	82.0 ± 4.2e	16.9 ± 2.3c	4.1 ± 0.8b	547 ± 90g
4CP	97.7 ± 8.2d	23.0 ± 2.4ab	7.0 ± 1.1ab	985 ± 105f
6CP	99.8 ± 7.7d	22.9 ± 1.6ab	9.8 ± 1.8ab	1135 ± 102d
8CP	100.7 ± 7.7d	24.3 ± 2.6a	10.4 ± 2.2ab	1522 ± 132cd
10HA	114.1 ± 5.0ab	23.9 ± 2.5ab	9.9 ± 1.2ab	1347 ± 90de
12HA	108.4 ± 4.5bc	22.7 ± 1.9ab	10.7 ± 1.8a	1402 ± 110d
15HA	100.4 ± 3.6d	21.5 ± 1.7b	9.9 ± 1.7ab	1387 ± 107d
4CP + 10HA	102.3 ± 3.0cd	22.9 ± 2.4ab	10.2 ± 2.7ab	1632 ± 90bc
6CP + 12HA	101.2 ± 2.4d	21.7 ± 1.9b	11.1 ± 2.4a	1772 ± 115b
8CP + 15HA	117.3 ± 4.4a	24.2 ± 2.5a	11.9 ± 2.0a	2135 ± 122a

Treatments: CK (Control, no amendments), 4CP (4 Mg ha^−1^ compost), 6CP (6 Mg ha^−1^ compost), 8CP (8 Mg ha^−1^ compost), 10HA (10 kg ha^−1^ humic acid), 12HA (12 kg ha^−1^ humic acid), 15HA (15 kg ha^−1^ humic acid), 4CP + 10HA (4 Mg ha^−1^ compost + 10 kg ha^−1^ humic acid), 6CP + 12HA (6 Mg ha^−1^ compost + 12 kg ha^−1^ humic acid) and 8CP + 15HA (8 Mg ha^−1^ compost + 15 kg ha^−1^ humic acid). Different letterings within a column exhibit substantial differences among the treatments and CK at a 5.0% probability level. Each value is Mean ± SD (n = 3).

## Discussion

In general, the application of organics such as compost and humic acid substantially increases the yield and production of winter field peas in arid soil. In this study, microbial C biomass, microbial N biomass, and microbial P biomass significantly increased when compost and humic acid were added as organic amendments to the arid soil. This increase led to a higher yield as well as an increase in the shoot and root biomass of winter field peas. This may be due to the microbial biomass providing sufficient mineral nutrition and space for the pea's root growth and development. Our results are in line with those of^22^, who reported a linear relationship between soil microbial communities and compost increments in the soil. Similarly^23^, also assessed an increase in microorganisms in soils by applying compost.

The winter field peas showed increased access to N and P when compost and humic acid were applied together, resulting in the highest N microbial biomass. The addition of compost and humic acid during the trial period likely facilitated quick absorption and colonization of microorganisms, followed by a delayed release of nutrients, which is likely the cause of this increase in bioavailability. However, it is currently unknown whether additional phosphorus is produced by the mineralization of extinct microbes or gradual excretion by soil biomass, which would subsequently raise the C:P ratio of the biomass of microbes. The microbial biomass serves as a significant source and sinks for N and P. Additionally, the shoot length and pod yield of winter field peas also increased substantially with the application of compost and humic acid, either as a single dose or in combination. Our findings are consistent with those of^24^, who observed an improvement in pea production and quality after the application of compost made from button mushroom waste substrate.

The combined incorporation of compost and humic acid at a rate of 8CP + 15HA resulted in the longest root length for the field peas, while the CK showed the shortest root length. Our findings support those of^25^, that who showed a rise in the root length and yield of mustard (*Brassica compestris* L.) cultivar S–9 when humic acid 6.35 kg acre^−1^ was incorporated at the time of cultivation in alkaline-calcareous soil. Similarly, the number of grains pod^-1^ and pod yield showed a maximum increase with the combined application of compost and humic acid with 8CP + 15HA. Our findings confirmed that of^26^, who described an increase in the pod's yield of winter peas when fertigated with phosphorus and inoculated with Rhizobium + PSB biofertilizer under subtropical conditions. The number of grains pod^−1^ was significantly increased by all the tested treatments except CK. In our experiments, the compost and humic acid incorporation into the arid soil resulted in enhanced winter peas’ nutrient absorption, microbial colonization, and soil microbial biomass indices. Our outcomes confirmed the results of^27^, who described that with the use of organic fertilizer (horse manure plus compost made from garden cuttings and shrubs) in an organic farming system, winter field pea yield increased as a result of increased nutrient absorption and microbial biomass in the soil.

Green pod yield was our primary objective for winter field pea cultivation. It was to be markedly affected by the application of compost and humic acid. The total green pod yield were substantially improved by increasing the amount of compost and humic acid. It may restore and rehabilitate the nutrition and quality of the arid soil in our experiments. Maximum green pod yield of 789.10 kg ha^−1^ and 798.36 kg ha^−1^ in both experiments was recorded with 8CP + 15HA. Our outcomes are inlined with those of^28^, who claimed that compost, straw, and coal ash additions boosted the production of barley and field peas while restoring the degraded soil. According to^28^, compost had the maximum advantages for enhancing soil quality and crop productivity.

The maximum shoot N, P, and K contents were recorded when organic amendments of 8CP + 15HA were applied. This may be due to compost being a natural component that contains many of the nutrient elements, including N, which is essential for plant growth. Humic acid and compost amendments resulted in a rise in shoots K, N, and P, which can be attributed to better nutrient usage efficiency due to the gradual release of nutrients and decreased losses. Our results support^29^, those who claimed that increasing the applied rates of compost and bio-compost on sandy soil significantly enhanced the vegetative development, yield, N, P, K, Mn, Fe, and total carbs contents of the pea.

Humic acids have been shown to have a potent synergistic effect with soil microbes, particularly for Bacillus spp. and Pseudomonas bacteria that increases plant growth, chlorophyll content, and nutrient absorption^30, 31^. The synergistic impact in our investigation when humic acid was paired with compost, notably in enhancing P uptake. The complex link between humic acid and P can be mediated by soil microbes. Bioactive chemicals like humic acid may cause changes in root morphology and architecture, enabling an expansion of the size and number of root systems and root hairs^32^, as well as extending bacterial association with the surface of the roots and enhancing the bacteria's persistence and activity^33^. These structural interactions between bacteria and plants in the presence of humic acid and compost might be the cause of the amplified benefits on P uptake for the microbial inoculants and humic acid combination seen in our study. Additionally, a coordinated action between humic acid and microbial inoculants that involves the lndole-3-acetic acid (IAA) biosynthesis pathway and may impact P availability has been documented^34^. The release of precursors IAA molecules such as L-tryptophan, which is an IAA precursor in both plants and microorganisms, as a result of increased root exudation may lead to localized acidification and the release of phosphate that has been solubilized in soil solution^35^.

Crop production in arid soils often faces a deficit in annual rainfall under no or limited artificial irrigation. Therefore, under reduced moisture content and low organic matter, arid soil produces low crop yields. In our experiments, the application of organic amendments such as compost and humic acid in arid soil substantially improved the growth and yield of winter field peas. Our results supported^36^, who reported that a feasible way to respond to the detrimental impact of water scarcity might be to supply organics to the soil in a reduced or deficit irrigation situation. Organic amendments retain and hold moisture for a longer period of time^36^. The organic incorporations in the form of compost of 10 t ha^−1^ and 5 t ha^−1^ substantially enhanced production for peas by 24 and 11% and by 41 and 25%, in full irrigation and water-deficient irrigation, respectively. Under situations of reduced moisture availability (like arid or semi-arid conditions), organic amendments in the soil dramatically increased yield and crop biomass output. Crop production may be enhanced by combining compost-like biostimulants with deficient moisture^36^. According to ^37^, improving the stress resilience of crops by adopting organic and natural resources is a pivotal approach for competing global food security.  

## Conclusio n

This study concluded that in arid soil, the addition of compost and humic acid as organic amendments substantially enhanced biomass production and yield of winter field peas by modulating soil microbial biomass and densities. The optimal combination of compost and humic acid in a suitable proportion of 8 Mg ha^−1^ compost + 15 kg ha^−1^ humic acid lowered the practice of chemical fertilizer use and enhanced winter field pea production. The incorporation of humic acid and compost is a practicable solution to counter detrimental impacts and rehabilitate nutrient-deficient weak arid soils to enhance legume crop productivity like field pea. As a prospect, it would be beneficial to explore whether a further increase in the rate of compost and humic acid beyond 8 Mg ha^−1^ compost + 15 kg ha^−1^, respectively, could either enhance or negatively impact winter field peas production in weak arid soil.

## Data Availability

All data generated and analyzed during this study are included in this published article.
